# Ferulic acid derivatives block coronaviruses HCoV-229E and SARS-CoV-2 replication in vitro

**DOI:** 10.1038/s41598-022-24682-9

**Published:** 2022-11-24

**Authors:** Sébastien Pasquereau, Mathilde Galais, Maxime Bellefroid, Irene Pachón Angona, Stéphanie Morot-Bizot, Lhassane Ismaili, Carine Van Lint, Georges Herbein

**Affiliations:** 1 Pathogens and Inflammation/EPILAB Laboratory, EA 4266, Université de Franche-Comté, Université Bourgogne Franche-Comté (UBFC), Besançon, France; 2 Service of Molecular Virology, Department of Molecular Biology (DBM), Université Libre de Bruxelles (ULB), 6041 Gosselies, Belgium; 3 Neurosciences Intégratives et Cliniques EA 481, Pôle de Chimie Organique et Thérapeutique, Univ. Bourgogne Franche-Comté, UFR Santé, Besançon, France; 4 Apex Biosolutions, Besançon, France; 5 Department of Virology, CHU Besançon, Besançon, France

**Keywords:** Virology, Viral infection, Drug discovery

## Abstract

A novel coronavirus, SARS-CoV-2, emerged in China at the end of 2019 causing a large global outbreak. As treatments are of the utmost importance, drugs with broad anti-coronavirus activity embody a rich and rapid drug discovery landscape, where candidate drug compounds could be identified and optimized. To this end, we tested ten small-molecules with chemical structures close to ferulic acid derivatives (FADs) (n = 8), caffeic acid derivatives (CAFDs) (n = 1) and carboxamide derivatives (CAMDs) (n = 1) for their ability to reduce HCoV-229E replication, another member of the coronavirus family. Among these ten drugs tested, five of them namely MBA112, MBA33, MBA27-1, OS4-1 and MBA108-1 were highly cytotoxic and did not warrant further testing. In contrast, we observed a moderate cytotoxicity for two of them, MBA152 and 5c. Three drugs, namely MBA140, LIJ2P40, and MBA28 showed lower cytotoxicity. These candidates were then tested for their antiviral propreties against HCoV-229E and SARS-CoV2 replication. We first observed encouraging results in HCoV-229E. We then measured a reduction of the viral SARS-CoV2 replication by 46% with MBA28 (EC50 > 200 µM), by 58% with MBA140 (EC50 = 176 µM), and by 82% with LIJ2P40 (EC50 = 66.5 µM). Overall, the FAD LIJ2P40 showed a reduction of the viral titer on SARS-CoV-2 up to two logs with moderate cytotoxicity which opens the door to further evaluation to fight Covid-19.

## Introduction

Human coronaviruses (HCoV) are positive-stranded RNA pathogens that are known to be rapidly evolving viruses. The coronaviridae family encompasses six different strains infecting humans divided into two genera: alphacoronavirus (alphaCoV) and betacoronavirus (betaCoV)^1^. The low pathogenic HCoV-229E and HCoV-NL63 strains are part of the alphaCoV. The betaCoV genera ramifies into the A lineage strains, including the more pathogenic HCoV-OC43 and HCoV-HKU1, and into the B and C lineage, respectively including the highly pathogenic severe acute respiratory syndrome coronavirus (SARS-CoV) and SARS-CoV-2, and the Middle East respiratory syndrome coronavirus (MERS-CoV)^2,3^. The lower and moderately pathogenic strains are endemic in the human populations, where they account for 15–30% of mild, self-limiting respiratory infections. These strains, namely HCoV-229E, HCoV-NL63, HCoV-OC43, and HCoV-HKU1, still present a greater incidence of lower respiratory tract infection in immunocompromised individuals^4^. The highly pathogenic strains include both the 2002 SARS-CoV and the 2012 MERS-CoV, which are linked to acute respiratory distress syndrome (ARDS). This syndrome potentially engender a chronic reduction in lung function, arrhythmia, or death^5^, with a mortality rate of 9.6% for SARS-CoV and 35.5% for MERS-CoV. SARS-CoV-2, the etiological agent behind Covid-19 disease, emerged in December 2019 in the city of Wuhan in China. After its pandemic outbreak, it became a major public health concern, inducing worldwide emergency measures^6^. While the average symptoms of infection are mild compared to SARS-CoV and MERS-CoV, SARS-CoV-2 can result in severe symptoms including hyperinflammation, cytokine storm and multiple organ complications. It can ultimately lead to death for some individuals^7^.

Aside from fastly developed vaccines^8^, very few effective curative therapy is available against SARS-CoV-2. To date, the recently developed molnupinavir and the new combination of nirmatrelvir and ritonavir have shown the most promising effect on SARS-CoV-2. In addition to these drugs, the use of dexamethasone, tocilizumab and baricitinib has been showed to improve the mortality in hospitalized patients with COVID-19^9–12^. Several drugs were previously used as curative therapies, but their efficiency were limited^13^. The development of new curative drugs is desperately needed^14^. It is well-known that it might take years for newly developed drugs to be clinically approved and reach the market^15^, despite the tremendous efforts put into their development. Repurposing existing drugs could be more effective than de novo drug discovery, with a reduced cost and time. A rapid evaluation of in vivo and in vitro efficacy is possible for existing drugs^16^. Additionally, drugs that were shown to inhibit the replication of other coronaviruses, including HCoV-229E, could potentially inhibit the replication of SARS-CoV-2 and other betacoronaviruses^17^.

Polyphenols have recently become an important group of compounds for the discovery of new drugs against human diseases^18^, including against Covid-19, as some recent studies already reported^19^. Recently we reported that resveratrol, a well-known nutraceutical with a multitude of biological properties and therapeutic applications belonging to polyphenols’ stilbenoids group^20^, inhibits the replication of both HCoV-229E and SARS-CoV-2^21^. Resveratrol is known for its anti-inflammatory activity^22^, and for its anti-aging and antioxidant properties^23^. Resveratrol showed a potential as an anti-viral agent against some RNA and DNA viruses. Possible target RNA viruses include influenza virus^24^, Zika virus^25^, rhinovirus^26^, rotavirus^27^ and MERS-CoV^28^, and DNA viruses including poxvirus^29^ and polyomavirus^30^.

Therefore we decided to assess the anti-coronavirus activity of other phenolic compounds, namely ferulic acid derivatives (FADs) and one caffeic acid derivative (CAFD), in addition to a carboxamide derivative (CAMD). Phenolic compounds display antiviral activity against herpes simplex virus-1 and rabies virus^31^. Ferulic acid can be extracted from various fruits and cereals and is, with its derivatives, a phenolic compound. Ferulic acid derivatives have been shown to inhibit H1N1 influenza virus replication in vitro^32^. Caffeic acids are found in coffee, fruits, and vegetables, and constitute one of the abundant plant-based phenols, which structures include 2 phenolic hydroxyl moieties. Caffeic acid and its derivatives previously showed an antiviral activity against herpes simplex virus^33^, SFTS (severe fever with thrombocytopenia syndrome) virus^34^, and influenza virus^35^. In addition, like resveratrol, phenolic compounds including FADs and CAFDs exhibit potent antioxidant and anti-inflammatory properties^36^. Carboxamide derivatives have been shown to inhibit influenza virus, parainfluenza virus type 3, norovirus, SARS-CoV and recently SARS-CoV-2 replication in vitro^37–41^. Therefore we tested the anti-coronavirus activity of MBA140, a carboxamide derivative newly synthesized, which displays the closest chemical structure to an oxazole-carboxamide derivative previously reported as efficient against SARS-CoV^41^.

In the light of what was mentioned previously, we screened ten molecules with chemical structures close to already reported molecules with anti-SARS-CoV-2 protease activities^42^ and belonging to the ferulic acid derivatives, caffeic acid derivatives, and carboxamide derivatives. For any of these drugs we tested in vitro the inhibitory effect on HCoV-229E and SARS-CoV2 replication.

## Materials and methods

### Viruses and reagents preparation

HCoV-229E was isolated from nasal and throat swabs collected from a man with mild upper respiratory illness (Human Coronavirus 229E ATCC VR-740). HCoV-229E stock virus (5 log PFU/mL) was propagated using MRC5 cells (RD Biotech, Besançon, France). MRC5 cells were cultured in DMEM media supplemented with 10% FCS. The infectious clone SARS-CoV-2 mNeonGreen (icSARS-CoV-2-mNG) strain was provided by Dr. Pei Yong Shi from University of Texas Medical Branch (UTMB)^43^. SARS-CoV-2-mNG stock virus (7 log TCID50/mL) was propagated in Vero E6 cells (ATCC) in DMEM media supplemented with 2% FCS. Viral titers were determined by endpoint dilution assays on Vero E6 cells and stored at − 80 °C until use.

### Drug preparation

The ten small molecules tested were synthetized at the University of Franche-Comté. As described previously^44–47^, the molecules were synthetized using the Ugi-4MCR method, starting from ferulic acid, caffeic acid or carboxamide. The resulting compounds were purified using flash column chromatography. After purification, drugs were stored in solid form at − 20 °C before preparation. Drugs were diluted in DMSO at a concentration of 10 mM, based on their molecular weight. The 10 mM stock was further diluted in cell culture media at final assay concentration at the time of use (1, 5, 10, 50, 100 or 200 µM).

### Viral replication assay

MRC5 cells were infected with HCoV-229E at MOI = 1 and treated at time of infection with compounds diluted in culture media during a 2-h incubation period. After removing the inoculum the cells were overlaid with culture media containing diluted compounds. Supernatants were collected after a 48-h incubation at 37 °C, to quantify viral loads by RT-qPCR and by plaque forming unit (PFU) assay as previously described^21,48,49^. For RT-qPCR, RNA was extracted using an RNA extraction kit (EZNA Total kit I, Omega BIO-TEK, Norcross, GA). RT-qPCR was performed based on a previously described protocol^49^, adapted using Superscript IV RT kit (ThermoFisher) for cDNA synthesis, followed by qPCR using KAPA SybrFast kit (KAPA Biosystems, KK4601) and the following primers : Forward 5′-TTCCGACGTGCTCGAACTTT-3′; Reverse 5′-CCAACACGGTTGTGACAGTGA-3′. The RT-qPCR assays were performed in duplicates of three independent experiments. For PFU assays, supernatants were used to infect MRC5 cells for 2 h. Cells were then overlaid with culture media containing agarose (1%). After a 48-h incubation, plaque forming units were counted under a light microscope using MTT staining of the cells. The PFU assays were performed in triplicates of a single experiment.

Vero E6 cells were treated with compounds at time of infection by SARS-CoV-2-mNG at MOI = 0.01 in DMEM supplemented with 2% FCS. The viral inoculum was removed after a 2-h incubation and the cells were overlaid with fresh DMEM media supplemented with 10% FCS containing the diluted compounds. After a 48-h incubation at 37 °C, supernatants were collected and frozen at − 80 °C prior to quantification and titration by RT-qPCR and endpoint dilution assay (TCID50/mL). RT-qPCR were performed on the collected supernatants. RNA was extracted from supernatent using RNA extraction kit QiAmp viral RNA kit (Qiagen ID 52906). RTqPCR was performed using TaqMan Fast Virus 1-Step MasterMix (ThermoFisher 4444432) and published SARS-CoV2 probes^50^: Forward (IDT 10006888); Reverse (IDT 10006890) Probe (IDT 10006892). Three independent replication assays were performed in triplicates for these viral replication assays. Classic tissue culture infectious dose 50% (TCID50/mL) in Vero E6 cells were performed in triplicates on viral stocks and on collected samples using the Reed & Muench statistical method.

### Cytotoxicity assays

MRC5 cells were treated with compounds for 48 h and MTT assay was performed as reported previously^21^. Cells were incubated with MTT reagent (1.2 mM, Life Technologies, Eugene, OR, USA) for 2 h. Acidic isopropanol was used to dissolve the formazan crystals and cell viability was measured by a spectrophotometer (Bio-Rad, Hercules, CA, USA). WST-1 viability assay was performed on Vero E6 cells treated for 48 h with compounds, following the manufacturer’s instructions (Roche Diagnostics GmbH, Mannheim, Germany). The cytotoxicity assays were performed in triplicates.

### Effective concentrations, cytotoxic concentrations and selectivity index calculations

The 50% effective concentrations (EC50), defined as a compound’s concentration inhibiting viral replication by 50%, was determined using a four- or three-parameter logistic regression (AAT Bioquest IC50 Calculator) to fit the dose–response curves. The cytotoxic concentration 50 (CC50), defined as a compound’s concentration reducing cell viability by 50%, was determined for each compound and cell type by the same method (AAT Bioquest IC50 Calculator). When our measurements did not reach 50% toxicity or 50% inhibition by studied concentrations, we used the estimated EC50 or CC50 value for further calculation of Selectivity Index (indicated as > 200 in Tables 2, 3). The Selectivity Index (SI) was calculated as previously reported^21^ by dividing the CC50 by the EC50 of every compound for each virus tested. A low SI (< 1) indicates a poor candidate for further development in therapeutical antiviral strategies. A high selectivity index (SI > 1) reflects an important range of concentrations that show an effective antiviral effect with low toxicity.

### Statistical analysis

RTqPCR experiments were performed in replicates as indicated. Values are presented as means of these independent experiments. Statistical significance or *p* value < 0.05 (represented by *) was assessed by performing the Wilcoxon signed-rank (value of 100). *P* value < 0.01 (**) or *p* value < 0.001 (***) were also represented. This analysis was performed using Graph Pad Prism 6 software.

#### .

## Results

The ten small molecules tested were synthesized at the University of Franche-Comté (Table 1), and were initially assessed as potential treatment for the oxidative stress associated with Alzheimer’s disease^44–47^. These molecules were selected based on their structure similarities with already reported inhibitors of nsp5 protease of SARS-CoV-2^42^. Interestingly these compounds have chemical structures close to ferulic acid derivatives (FADs), caffeic acid derivatives (CAFDs) and carboxamide derivatives (CAMDs) (Table 1)^44–47^. Among the ten compounds, eight were FADs, namely LIJ2P40, OS4-1, MBA108-1, MBA33, MBA152, 5c, MBA112 and MBA28. MBA27-1 is a CAFD and MBA140 is a CAMD.

**Table 1:** The ten small-molecules tested belonging to the FADs, the CAFDs or the CAMDs.

FAD		FAD	
Structure	Name	Structure	Name
	LIJ2P40		OS4-1
	MBA 108-1		MBA 33
	MBA 152		MBA112
	5c		MBA28

CAFD		CAMD	
Structure	Name	Structure	Name
	MBA27-1		MBA140

**Figure 1:**
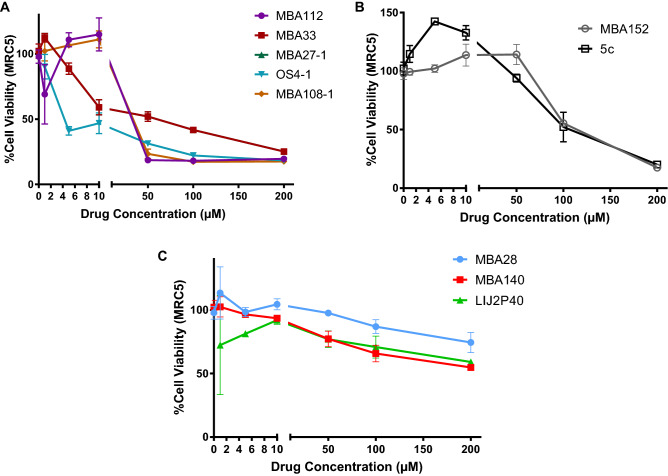
Viability of MRC5 cells under treatment with the ten drugs using MTT toxicity assay. (**A**) High cytotoxicity was observed for MBA112, MBA33, MBA27-1, OS4-1 and MBA108-1, (**B**) intermediate cytotoxicity for MBA152 and 5c and (**C**) low cytotoxicity for MBA140, LIJ2P40, and MBA28. MTT assays were performed after 48 h. Untreated cells were used for normalization of MTT assay. Results are represented as mean ± SD of three independent experiments.

Among these ten small molecules tested, five, MBA112, MBA33, MBA27-1, OS4-1 and MBA108-1, showed high cytotoxicity on MRC5 cells with a CC50 of 35.5 µM, 39.4 µM, 22.5 µM, 8.0 µM and 34.4 µM respectively (Fig. 1A, Table 2). In addition, two molecules MBA152 and 5c displayed moderate cytotoxicity with a CC50 of 108.3 µM and 89.0 µM respectively (Fig. 1B, Table 2). By contrast, three molecules MBA140, LIJ2P40, and MBA28 showed low cytotoxicity on MRC5 cells with a CC50 above 200 µM (Fig. 1C, Table 2), and were further tested.

**Table 2:** CC50 on MRC5 cells of the ten drugs studied for their toxicity.

Drug tested	CC50 (µM)
MBA112	35.5
MBA33	39.4
MBA27-1	22.5
OS4-1	8.0
MBA108-1	34.4
MBA 152	108.3
5c	89.0
MBA28	> 200
MBA140	> 200
LIJ2P40	> 200

**Figure 2:**
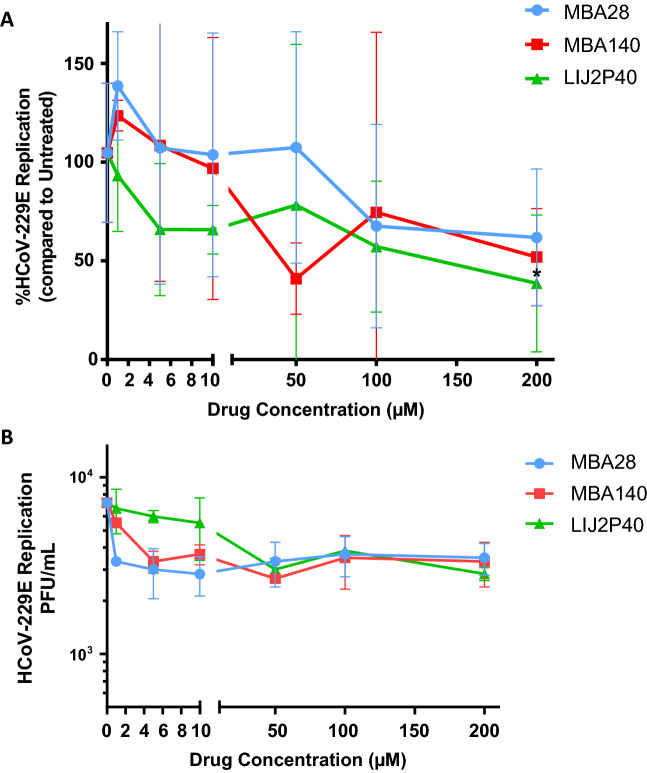
Inhibition of replication of high HCoV-229E viral load measured by RT-qPCR (**A**) and PFU assay (**B**) on MRC5 cells by LIJ2P40, MBA140 and MBA28. Cells were treated by compounds at the time of infection with HCoV-229E (MOI = 1). PFU assay and RT-qPCR were performed after 48 h. Untreated cells infected with HCoV-229E (864.10^4^ copies/mL) were used for normalization. RT-qPCR results are represented as mean ± SD of three independent experiments done in duplicates. PFU assay results are represented as mean ± SD of a single experiment done in triplicates. The Wilcoxon signed-rank test (value of 100) was used as statistical test with *P* ≤ 0.05 = *.

The three less cytopathic drugs (MBA28, MBA140 and LIJ2P40) were tested for their ability to inhibit viral replication of HCoV-229E in MRC5 cells. Cells were infected by HCoV-229E MOI = 1 during two hours in presence of various concentrations of each drug (0.1, 5, 10, 50, 100 or 200 µM). After infection, cells were cultured in presence of treatment or not and viral replication was analyzed in supernatant after 48 h either by RT-qPCR (Fig. 2A) or Plaque Forming Unit assay (PFU) (Fig. 2B). Taken together, our data suggest a decreasing viral replication, measured by RT-qPCR, with all three drugs (Fig. 2A). We were also able to observe only a moderate inhibition of replication of HCoV-229E by PFU assays at higher concentrations (> 50 µM) (Fig. 2B).

**Figure 3:**
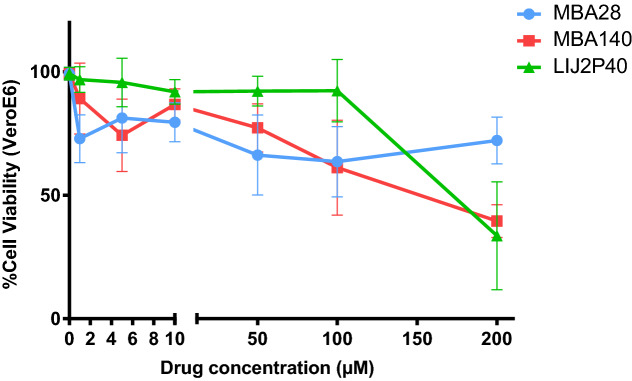
Viability of Vero E6 cells treated with MBA28, MBA140 or LIJ2P40 drugs measured by WST-1 assay. WST-1 viability assays were performed after 48 h. Untreated cells were used for normalization of the WST-1 viability assay. Results are represented as mean ± SD of three biological replicates.

Based on the preliminary results obtained using the HCoV-229E model, we also tested the ability of MBA28, MBA140 and LIJ2P40 to inhibit viral replication of SARS-CoV-2-mNG in VeroE6 cells. First, the cytotoxicity of LIJ2P40, MBA140 and MBA28 on Vero E6 cells was tested by WST-1 (Fig. 3). Results showed that below 50 µM, all three drugs MBA28, MBA140 and LIJ2P40 showed a moderate toxicity, with a CC50 above 200 µM for MBA28, a CC50 of 174.3 µM for MBA140 and a CC50 of 192.6 µM for LIJ2P40 (Table 3). Higher concentrations (200 µM) showed a moderate effect on VeroE6 cells for MBA28 and more important toxicity for MBA140 and LIJ2P40.

**Table 3:** EC50, CC50 and SI for the 3 drugs tested for SARS-CoV-2 replication in VeroE6 cells.

Drug tested	CC50 (µM)	EC50 (µM)	SI
MBA 28	> 200	> 200	< 1
MBA 140	174.3	176.3	1
LIJ2P40	192.6	66.5	2.9

**Figure 4:**
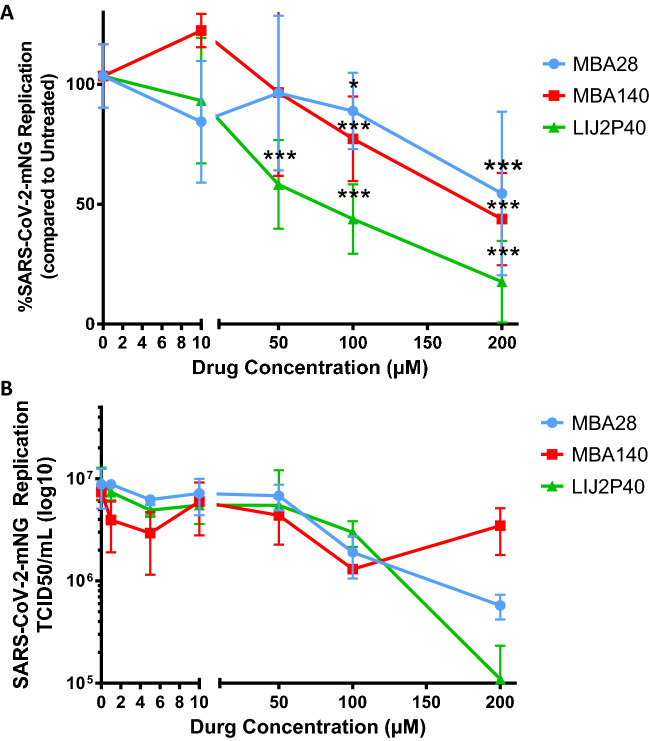
Inhibition of SARS-CoV-2-mNG replication in VeroE6 cells measured by RT-qPCR **(A)** and endpoint dilution (TCID50/mL) **(B**), for LIJ2P40, MBA140 and MBA28. Cells were treated by compounds at the time of infection with SARS-CoV2-mNG (MOI = 0.01). Endpoint dilution assays and RT-qPCR were performed 48 h post-infection. Untreated cells infected with SARS-CoV2-mNG (527.10^6^ copies/mL) were used for normalization of the assay. RT-qPCR results are represented as mean ± SD of three independent experiments done in triplicates. TCID50 results are represented as mean ± SD of a single experiment done in triplicates. The Wilcoxon signed-rank test (value of 100) was used as statistical test with *P* ≤ 0.05 = *; *P* ≤ 0.01 = **; *P* ≤ 0.001 = ***.

The moderate toxicity of tested compounds further encouraged us to test their antiviral properties of SARS-CoV2-mNG by performing infections at MOI = 0.01 during two hours on VeroE6 cells in presence of treatment or not. Cells were then cultured during 48 h in presence of various concentrations of each drug (0, 1, 5, 10, 50, 100 or 200 µM), and viral replication was analyzed in supernatant either by RT-qPCT (Fig. 4A) or endoint dilution assays (TCID50) (Fig. 4B). We observed a significant decrease in SARS-CoV-2-mNG replication under MBA28 treatment at concentrations of and above 100 µM, with a 46% maximal inhibition at 200 µM (Fig. 4A). For MBA140 treatement, we observed a similar significant decrease in SARS-CoV-2-mNG replication at concentrations starting at 100 µM, with a 58% maximal inhibition (Fig. 4A). By contrast, the maximal percentage of inhibition of SARS-CoV-2-mNG reached 82% with an LIJ2P40 concentration of 200 µM. We also observed a significant decrease in SARS-CoV-2-mNG replication starting at 50 µM of LIJ2P40, with a 42% decrease of viral replication measured by RT-qPCR (Fig. 4A). The inhibition of SARS-CoV-2-mNG replication observed in cells treated with LIJ2P40 did not follow the reduction in cell viability and was a genuine anti-SARS-CoV-2 effect of this drugs in vitro.

Inhibition of SARS-CoV2-mNG replication was also observed by TCID50 assay at concentrations > 100 µM for MBA28 and LIJ2P40 (Fig. 4B). Overall, among the three drugs tested, LIJ2P40 showed the highest anti-SARS-CoV-2 activity.

Based on these results, we measured an EC50 above 200 µM for MBA28, of 176.3 µM for MBA140 and LIJ2P40 showed the lowest EC50 at 66.5 µM (Table 3). We calculated the selectivity index for the inhibition of SARS-CoV-2-mNG in Vero E6 cells for the three drugs tested. Both MBA28 and MBA140 present a low SI, both below or equal to 1 (Table 3). The SI for LIJ2P40 is higher, above 2.9 (Table 3) showing an interesting potential for further testing in other models and potentially therapeutic antiviral strategies against coronaviruses infections.

## Discussion

We showed the antiviral effect of ferulic acid derivatives, notably MBA28, MBA140 and LIJ2P40, on HCoV-229E and SARS-CoV-2 replication in vitro. These drugs showed a decrease in HCoV-229E replication, as measured by RT-qPCR. However, the important variation between biological replicates did not allow us to conclude based on these data. The SD values obtained were still very important due to difficulties in the technical replication of the experiments. As the experimental conditions were not entirely optimized and the sensitivity of the assay was not as good as it should be, we decided not to conclude on these data. In addition, the assessment of the compounds effect on HCoV-229E replication is an intermediate step in our testing pipeline, where SARS-CoV-2 inhibition is our main focus.

The overall efficiency of these three drugs against HcoV-229E is too limited to warrant their use as is, but they could be good candidates as base molecules to be modified for enhanced antiviral effect. We however considered them having enough potential to be further tested on SARS-CoV-2.

We observed a moderate cytotoxicity of MBA28 on Vero E6 cells, with a CC50 of > 200 µM and 174.3 µM for MBA140. The testing of LIJ2P40 however showed a more gentle cytotoxicity at lower concentrations, however high concentration of 200 µM showed important toxicity. Indeed the effect of the solvent used to solubilize the compounds (DMSO) are expected to impact the viability assays results, especially at high concentrations (2%). Additional testing of possible solvents and their effects should be performed and taken into account for pursuing more advanced antiviral testing. Also, a small increase in viability at the highest concentration (200 µM) was globally observed for MBA28, compared to 100 µM. This could be the results of the antioxidant properties of the molecule^36^. Since the viability was measured using WST-1 assay, an antioxidant could impact the tetrazolium reduction, which is measured by such assay^51^. This effect might be more pronounced at higher concentrations.

The moderate cytotoxicity measured in Vero E6 cells warranted the further testing of these three drugs for SARS-CoV-2 inhibition. We observed a decrease in SARS-CoV-2-mNG replication in vitro at high doses of MBA28 (EC50 > 200 µM) and at high doses of MBA140 (EC50 = 176.3 µM)*.* As we did note the TCID50 assay showed an increase in SARS-CoV-2-mNG replication at 200 µM dose of MBA140, compared to 100 µM, we do need to underline the limited number of replicates in these experiments. However, our RT-qPCR results, which is a more sensitive and reproducible method, and also based on triplicates of three independent experiments, showed a significant inhibition at those concentrations thereby providing strong evidence toward the antiviral effect observed. In addition, we found a significant inhibition of SARS-CoV-2-mNG replication with LIJ2P40, starting at 50 µM concentration (EC50 = 66.5 µM). Regarding LIJ2P40 treatment, TCID results, however poor in replicates, did show an interesting decrease of two logs at higher concentrations. While the toxicity at these concentrations was notable, the significant reduction of mean replication compared to untreated as measured by RT-qPCR showed an additional inhibition by 18%, demonstrating a genuine antiviral effect for this molecule. Therefore, of the three drugs tested against SARS-CoV-2-mNG, LIJ2P40 showed the most promising inhibition of SARS-CoV-2 replication, with limited toxicity. To further confirm the anti-SARS-CoV-2 effect of these drug using an in vitro model closer to the in vivo pathophysiology, we will assess their effect on SARS-CoV-2-mNG infections using human respiratory tract epithelial cell lines, for instance lung epithelial cell lines A549 and Calu or nasal epithelial cell line RPMI2650. Additionally, 3D organotypic nasal epithelial cultures (commercially available MucilAir™) could be used to better reflecting the pathophysiology of SARS-CoV-2 infection than the current macaque renal epithelial model of Vero E6 cells.

Polyphenols have been previously showed to be efficient against viral infections, by acting on the modulation of immune system^52^. They were also shown to directly inhibit viral replication^53^ or reduce viral entry^54^. CAFDs are phenolic compounds which possess prominent antiviral activity especially against hepatitis B virus (HBV) and HIV^55^. Recent docking analysis on CAFDs showed that they possessed high binding energies against the main protease Nsp5 (M^Pro^), Nsp15 and the S2 spike subunit of SARS-CoV-2. They could be potent modulators of Covid-19, since their binding energy is even higher than nelfinavir^19^.

FADs, as part of phenols, share properties with other polyphenols such as resveratrol. The observed antiviral effects could be linked to their interactions with both viral proteases (Nsp3 and Nsp5) but also with the spike protein. The phenol groups could allow the disruption of the enzymatic function of Nsp3 and Nsp5. The interaction with spike proteins could also prevent capsid formation, thus reducing the viral replication.

A similarity of FADs and CAFs chemical structure with previously reported small molecules^42^ could explain their potential as antiviral drugs. These small molecules were predicted to target M^Pro^ of SARS- and MERS-CoV, by the presence of hydrophobic regions. The disruption of M^Pro^ protease activity results in an important inhibition of viral replication^56^. CAMDs have been reported to display antiviral activity against RNA viruses including influenza virus, parainfluenza virus, norovirus, SARS-CoV and SARS-CoV-2^37–41^. Therefore we tested the anti-coronavirus activity of MBA140, a carboxamide derivative newly synthesized, which displays the closest chemical structure to an oxazole-carboxamide derivative previously reported as efficient against SARS-CoV^41^. Although MBA140 inhibits both HCoV-229E and SARS-CoV2 its antiviral effect was limited and precludes its further testing in preclinical models of SARS-CoV-2.

## Conclusions

The overall efficiency of the FAD compounds MBA28 and the CAMD compound MBA140 against SARS-CoV-2 is too limited to warrant their use as is, but they could be good candidates as base molecules to be modified for enhanced antiviral effect in the future. In addition, we show here the antiviral effect of the FAD LIJ2P40, both on HCoV-229E and SARS-CoV-2 replication in vitro*.* Our testing for SARS-CoV-2 inhibition shows a strong anti-viral effect of LIJ2P40 with moderate cytotoxicity in vitro. However, further testing in more physiological in vitro models of human respiratory tract epithelial cell lines and 3D organotypic nasal epithelial (MucilAir™) cultures is needed to confirm whether these compounds could be of use in a clinical setting, alone or in combination with other treatments. Finally, it is important to note that our results on SARS-CoV-2 inhibition are in vitro data and do not indicate that any of the drugs tested can be used to treat Covid-19 until double-blinded clinical assays have been successfully performed.

## Data Availability

The datasets used and/or analyzed during the current study are available from the corresponding author on reasonable request.
